# Awareness of Carpal Tunnel Syndrome Among Adults in the Western Region of Saudi Arabia: A Cross-Sectional Study

**DOI:** 10.7759/cureus.42777

**Published:** 2023-07-31

**Authors:** Abdullah Tawakul, Mohammed H Alharbi, Fadi S Althobaiti, Rayan A Bokhary, Mahmoud A Alsaadi, Abdulrahman A Almalki, Sayyaf Alhazmi, Nawaf Alhazmi, Hassan A Alhazmi

**Affiliations:** 1 Department of Medicine, Umm Al-Qura University, Makkah, SAU

**Keywords:** orthopedics, neurology, saudi arabia, western region, jeddah, medinah, taif, makkah, awareness, carpal tunnel syndrome

## Abstract

Introduction

Carpal tunnel syndrome (CTS) is a peripheral neuropathy that happens when the median nerve is compressed by the transverse carpal ligament within the carpal tunnel. Public awareness is crucial for early detection and intervention; therefore, this study aims to assess the awareness of CTS among the adult population in the western region of Saudi Arabia.

Methods

The study design was a cross-sectional study where 1400 participants (although 1199 answers were included) from the western region were randomly asked to fill in an online questionnaire that was delivered to them via social media applications.

Results

The study sample was predominantly females (57.1%), and 88.7% were Saudi, mostly students and professionals (53% and 25.2% respectively); predominant chronic illness among study participants was diabetes mellitus (7%), and only 2% of the sample (27 participants) were diagnosed with CTS. Participants showed more level of awareness regarding the causes and features of CTS, 630 participants (52.5%) had a good awareness of the causes, and 652 (54.4%) had a good awareness of the features. Meanwhile, participants showed a poorer level of awareness regarding treatment, effects, and prevention; percentages of poor awareness were 56% (672) for treatment, 51.9% (622) for effects, and 52.8% (633) for prevention.

Conclusion

The study shows that the adult population in the western region of Saudi Arabia had insufficient awareness of CTS, especially regarding treatment, effects, and prevention. Therefore, more campaigns should be made to enhance population awareness of CTS, and the study also suggests a link between CTS and chronic diseases.

## Introduction

Carpal tunnel syndrome (CTS) is the most common peripheral nerve entrapment syndrome worldwide. It occurs due to the compression of the median nerve in a space within the wrist called “carpal tunnel.” It is usually idiopathic, but it can be due to thickened ligaments, tendon sheaths, or enlarged bones. A typical history includes the patient waking up with numbness, tingling sensations, and pain in the median nerve distribution (the first three digits and the median half of the fourth digit). Wasting of the abductor pollicis brevis muscle develops with sensory loss in the radial three-and-a-half fingers [1,2]. Although ultrasound (US) and magnetic resonance imaging (MRI) can be used, nerve conduction studies are the most accurate test to diagnose carpal tunnel syndrome [3]. Splints are used to treat mild to moderate CTS symptoms conservatively, and local steroid injections can result in significant, transient relief [4]. However, for severe cases, surgery is indicated [5]. Multiple risk factors were associated with the development of CTS.

An estimated prevalence of 4% was observed in the general population in a Sweden study [6] and 14% in a recent Saudi study [7]. According to previous studies, female gender, ages between 41 and 60 years, diabetes mellitus, menopause, hypothyroidism, obesity, arthritis, and pregnancy are identified as risk factors for developing CTS [8,9]. Furthermore, CTS has various physical and financial implications in both short and long terms, involving absences from work, a decline in neurologic function, and significant healthcare expenses [10].

There are a few studies that were done to assess the awareness of CTS generally and locally. However, two recent studies were conducted locally to assess the prevalence and levels of awareness among the general population in Saudi Arabia. The first one was conducted in Al-Majmaah city, in which 386 participants were included. It reported that there was sufficient knowledge among the general population. Additionally, the study also demonstrates a prevalence of 14% and a significant association between CTS and chronic illnesses, such as diabetes mellitus, hypothyroidism, and rheumatoid arthritis [7]. As for the second study, it was conducted in the Al-Jouf region among 420 participants, which reported insufficient awareness among 74.8% with a significant association with age [11]. In addition, a study in the Netherlands among 715 participants concluded that the prevalence of CTS was 0.6% among males and 5.8% among females [12].

Unfortunately, there is no previous study to assess the awareness of CTS among adults in the western region of Saudi Arabia. Therefore, our aim in this study is to determine the levels of awareness of CTS among adults in the western region of Saudi Arabia, including Makkah, Taif, Jeddah, and Al-Medinah cities.

## Materials and methods

Study design

The adopted study design was a descriptive cross-sectional study with the use of an online questionnaire that has been used in a previous study [7] and a non-probability snowball sampling technique. The questionnaire consisted of four parts. The first part assessed sociodemographic characteristics (gender, age, nationality, city of residence, marital status, education level, occupation, and family monthly income); the second part measured the prevalence of chronic diseases associated with a high risk of developing CTS by asking participants whether they were clinically diagnosed of any (rheumatoid arthritis, hypothyroidism, diabetes mellitus, amyloidosis, or none); the third part measured the prevalence of CTS in which participants were asked if they have been clinically diagnosed with CTS, and the last part consisted of six multiple choice questions in which participants can select more than one answer (if the answer “I don’t know” was not chosen) to assess the awareness of different categories regarding CTS (causes, clinical features, treatment, effects, and prevention). Participants received the questionnaire through social media platforms, and the electronic data collection forms did not ask for any personal information.

Study population

Public adults who live in Saudi Arabia's western region and speak both Arabic and English are the target population. The study did not include individuals who did not complete the survey, live outside the western region, or did not speak Arabic or English.

Sampling methodology

This study sample was surveyed between March 1 and April 1, 2023, with all participants providing their consent. A sample size of 768 participants was suggested by OpenEpi version 3.1 when considering a 95% confidence interval level, an anticipated frequency percentage of 50%, and a design effect of 2 because of a large population.

Data analysis

The collected data were extracted, reviewed, coded, and then entered into BlueSky Statistics version 10.2.1 (BlueSky Statistics LLC, Chicago, IL). Frequencies and percentages were used to present the results. All variables, including sociodemographic factors, the prevalence of chronic diseases, the prevalence of CTS, and awareness of the causes, features, treatments, effects, and prevention of CTS, were subjected to descriptive statistics. After this, participants were divided into two groups based on their level of awareness in each category: good (if they chose at least one response) or poor (if they selected "I don't know"). The Chi-square test was additionally utilized to investigate the association between demographics and each category's awareness level as well as the prevalence of CTS and chronic illnesses. A 0.05 or lower p-value indicates a significant association.

Ethical part and confidentiality

Each participant was asked to provide consent via the questionnaire before participating in the study. The questionnaire included a clear description of the study's objectives, which was shown to the participants. To protect participants' privacy, their identities were kept confidential and anonymous, and only the research team had access to the responses. The study obtained ethical approval from the Biomedical Ethics Committee of Umm Al-Qura University (No: HAPO-02-K-012-2023-02-1483).

## Results

A total of 1400 responses were collected; 120 were excluded for being younger than 18 years old, and 81 were excluded as they were not from the western region of Saudi Arabia. Eventually, 1199 were included in our study as they fulfilled our inclusion criteria. Table 1 shows demographic information on 1,199 study participants, with 42.9% of whom are male and 57.1% of whom are female. Most of the participants were 18-25 years old (59%), were Saudi (88.7%), and lived in Makkah (32.7%), Jeddah (23.9%), Medinah (23.7%), and Taif (19.8%). The majority were single (62.2%), students (53%), had university-level education (66.2%), and had a monthly income of 10,001-15,000 SAR (24.2%).

**Table 1:** Sociodemographic characteristics of participants

Demographic data			
Variables		n = 1199	%
Gender	Male	514	42.9%
	Female	685	57.1%
Age	18-25 years	708	59%
	26-35 years	138	11.5%
	36-45 years	131	10.9%
	46-55 years	148	12.3%
	>55 years	74	6.2%
Nationality	Saudi	1064	88.7%
	Non-Saudi	135	11.3%
City of residence	Makkah	392	32.7%
	Jeddah	286	23.9%
	Medinah	284	23.7%
	Taif	237	19.8%
Marital status	Single	746	62.2%
	Married	411	34.3%
	Divorced	28	2.3%
	Widowed	14	1.2%
Education level	Primary	7	0.6%
	Intermediate	29	2.4%
	Secondary	308	25.7%
	University	794	66.2%
	Postgraduate	61	5.1%
Occupation	Student	636	53%
	Professional	302	25.2%
	Self-employed	24	2%
	Retired	82	6.8%
	Housewife	84	7%
	Unemployed	71	5.9%
Family monthly income	0-5000 SAR	257	21.4%
	5001-10000 SAR	223	18.6%
	10,001-15,000 SAR	290	24.2%
	15,000-20,000 SAR	218	18.2%
	>20,000 SAR	211	17.6%

**Figure 1:**
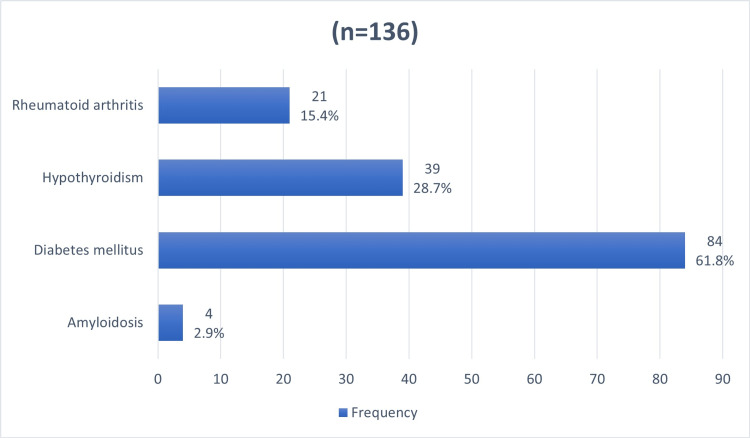
Prevalence of chronic diseases among participants

Figure 1 displays the frequency of chronic diseases among study participants, with 11.3% diagnosed with either rheumatoid arthritis (RA), hypothyroidism, diabetes mellitus (DM), or amyloidosis. Among those with these conditions, DM was the most prevalent, affecting 84 (7%) participants. Hypothyroidism and RA were less common, with 39 (3.3%) and 21 (1.8%) participants, respectively. Amyloidosis was the least common condition, with only four (0.3%) affected.

**Figure 2:**
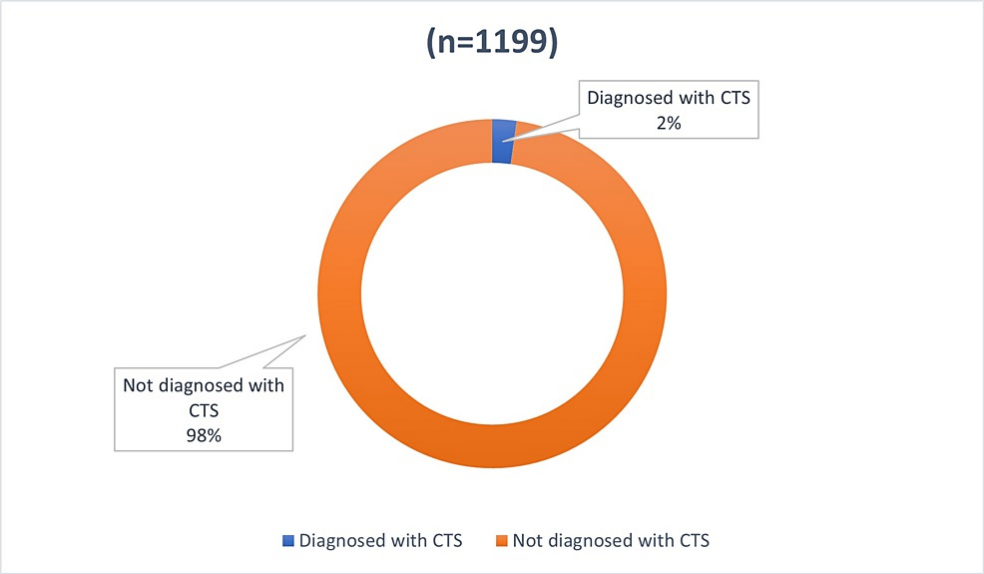
Prevalence of CTS among participants CTS: Carpal tunnel syndrome.

Figure 2 shows the prevalence of CTS among the study participants, with only 27 individuals (2%) being diagnosed with it. The remaining 1172 participants (98%) were not diagnosed with CTS.

Table 2 displays the frequency of awareness regarding the cause of CTS among respondents. Results show that the majority of participants, 569 (47.5%), were unaware of the causes of CTS. On the other hand, 630 (52.5%) respondents stated that they were aware of the causes. Among those who were aware, 408 (29.2%) recognized repeated physical activity as a potential cause. Arthritis was the second-most identified cause, with 322 (23.1%) participants recognizing it. Additionally, 137 (9.8%) participants identified tumors of the bone as a potential cause, while 320 (22.9%) and 208 (14.9%) identified wrist fractures or dislocations and trauma, respectively, as potential causes.

**Table 2:** Awareness frequency of the causes of CTS CTS: Carpal tunnel syndrome.

Awareness of the causes of CTS		
Variables	n	%
I don’t know	569	47.5%
I know	630	52.5%
Frequency of the identified causes		
Arthritis	322	23.1%
Tumor of bone	137	9.8%
Repeated physical activity like using a computer and taping	408	29.2%
Wrist fracture or dislocation	320	22.9%
Trauma	208	14.9%

Table 3 displays responses regarding awareness of the clinical features of CTS among participants. About 547 (45.6%) participants answered "I don't know" when asked about the clinical features of CTS, indicating a lack of awareness among almost half of the participants. A total of 459 (25.3%) participants who were aware of the clinical features acknowledged wrist pain, making it the most identified clinical manifestation of CTS, while 371 respondents (20.5%) mentioned tingling and numbness in the thumb, index, and middle fingers as clinical features of CTS. Some respondents identified other clinical features of CTS, such as weakness in the thumb muscle (235, 13%), reduced grip strength (259, 14.3%), and muscle wasting in the hand (215, 11.9%).

**Table 3:** Awareness frequency of the clinical features of CTS CTS: Carpal tunnel syndrome.

Awareness of the clinical features of CTS		
Variables	n	%
I don’t know	547	45.6%
I know	652	54.4%
Frequency of the identified clinical features		
Pain in wrist	459	25.3%
Changing pain intensity while moving the wrist	272	15%
Tingling and numbness in the thumb, index, and middle fingers	371	20.5%
Weakness affecting the thumb muscle	235	13%
Decreased overall hand grip	259	14.3%
Muscle wasting in the hand	215	11.9%

Table 4 displays the frequency of responses regarding the treatment of CTS. Only 527 (44%) of the participants could identify at least one treatment for CTS. Splinting and oral analgesics were the most identified treatments for CTS, with 340 (30.2%) and 313 (27.8%) of the participants being able to identify them, respectively. Non-steroidal anti-inflammatory drugs (NSAIDs) and steroid injections were also identified as potential treatment options by 268 (23.8%) and 204 (18.1%) respondents, respectively. However, most of the participants (672, 56%) answered "I don't know" when asked about the treatment options for CTS, indicating a lack of awareness among the respondents regarding the treatment of CTS.

**Table 4:** Awareness frequency of the treatment of CTS CTS: Carpal tunnel syndrome; NSAIDs: Non-steroidal anti-inflammatory drugs.

Awareness of the treatment of CTS		
Variables	n	%
I don’t know	672	56%
I know	527	44%
Frequency of the identified treatments of CTS		
NSAIDs	268	23.8%
Splint	340	30.2%
Steroid injection	204	18.1%
Oral analgesics	313	27.8%

Table 5 displays the awareness of the effects of CTS among the respondents. It shows that over half of the participants (622, or 51.9%) were not aware of the effects of CTS. However, 577 (48.1%) were able to identify at least one of the effects of CTS. Among those, 483 (51.1%) recognized that CTS could affect a patient's job performance. Only 268 (28.4%) recognized its impact on the patient's sleep, and 194 (20.5%) identified its effect on the patient's social life.

**Table 5:** Awareness frequency of the effect of CTS CTS: Carpal tunnel syndrome.

Awareness of the effect of CTS		
Variables	n	%
I don’t know	622	51.9%
I know	577	48.1%
Frequency of the identified effects of CTS		
CTS affects the patient's job performance	483	51.1%
CTS affects the patient's sleep	268	28.4%
CTS affects the patient's social life	194	20.5%

Table 6 displays the frequency of answers for preventive methods for CTS. About 566 (47.2%) participants were able to identify at least one of the preventive methods for CTS, with avoiding repetitive movements being the most identified method (340, 26.2%) followed by keeping the wrist straight while resting (300, 23.1%). Avoiding falls or direct trauma was recognized as a prevention method by 333 (25.6%) participants; wearing a splint while sleeping (186, 14.3%) and staying warm (140, 10.8%) were also identified as preventive measures. However, more than half of the participants (633, 52.8%) were not aware of any preventive measures for CTS.

**Table 6:** Awareness frequency of the prevention of CTS CTS: Carpal tunnel syndrome.

Awareness of the prevention of CTS		
Variables	n	%
I don’t know	633	52.8%
I know	566	47.2%
Frequency of the identified preventive methods of CTS		
Avoid falls or direct trauma	333	25.6%
Avoid repetitive movement	340	26.2%
Stay warm	140	10.8%
Keep your wrist straight while at rest	300	23.1%
Wear splint while sleeping	186	14.3%

**Figure 3:**
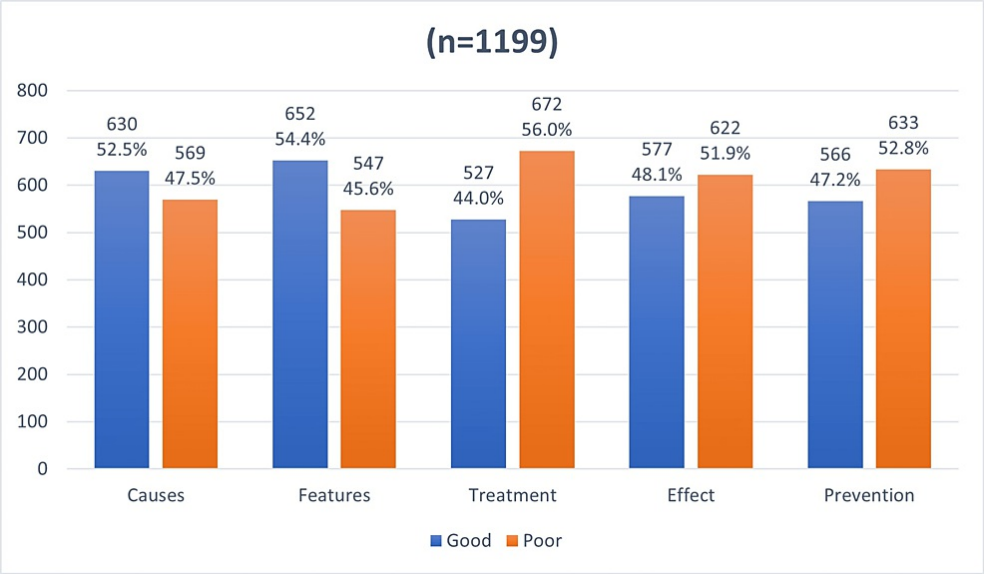
Overall levels of awareness toward CTS CTS: Carpal tunnel syndrome.

Figure 3 shows the overall awareness of study participants about CTS. The results show that 52.5% of respondents had a good level of awareness of the causes of CTS, while 47.5% had a poor level of awareness. For features of CTS, 54.4% of participants had a good level of awareness, and 45.6% had a poor level of awareness. However, for the treatment, effect, and prevention of CTS, the majority had a poor level of awareness, with 56%, 51.9%, and 52.8%, respectively.

Table 7 shows the level of awareness of CTS and its association with demographics. Females tend to have slightly better awareness than males across all aspects of CTS, with good awareness levels ranging from 45.8% to 54.7% for females and 41.4% to 53.9% for males. However, this difference is not statistically significant except for effect awareness, where it was found to be statistically significant (p = 0.02). Younger participants in the study tended to have higher levels of awareness about CTS compared to older participants, with good awareness levels ranging from 42.9% to 56.5% in younger age groups and 52.7% to 45.1% in older age groups. Interestingly, awareness about prevention slightly increased in older age groups. However, poor awareness levels were still present, ranging from 57.1% to 43.5% in younger age groups and 55.0% to 45.9% in older age groups, but these differences were not statistically significant.

**Table 7:** Association between the level of awareness and sociodemographic variables *P-value < 0.05 is statistically significant. **P-value < 0.01 is statistically highly significant.

Variables		Causes		Features		Treatment		Effect		Prevention	
Gender		Good	Poor	Good	Poor	Good	Poor	Good	Poor	Good	Poor
Male	n	258	256	277	237	213	301	227	287	231	283
	%	50.2	49.8	53.9	46.1	41.4	58.6	44.2	55.8	44.9	55.1
Female	n	372	313	375	310	314	371	350	335	335	350
	%	54.3	45.7	54.7	45.3	45.8	54.2	51.1	48.9	48.9	51.1
P-value		0.16		0.77		0.13		0.02*		0.17	
		Causes		Features		Treatment		Effect		Prevention	
Age		Good	Poor	Good	Poor	Good	Poor	Good	Poor	Good	Poor
18-25 years	n	368	340	400	308	304	404	339	369	322	386
	%	52.0	48.0	56.5	43.5	42.9	57.1	47.9	52.1	45.5	54.5
26-35 years	n	78	60	71	67	61	77	66	72	68	70
	%	56.5	43.5	51.4	48.6	44.2	55.8	47.8	52.2	49.3	50.7
36-45 years	n	65	66	66	65	60	71	59	72	62	69
	%	49.6	50.4	50.4	49.6	45.8	54.2	45.0	55.0	47.3	52.7
46-55 years	n	80	68	76	72	68	80	73	75	76	72
	%	45.1	45.9	51.4	48.6	45.9	54.1	49.3	50.7	51.4	48.7
>55 years	n	39	35	39	35	34	40	40	34	38	36
	%	52.7	47.3	52.7	47.3	45.9	54.1	54.1	45.9	51.4	48.6
P-value		0.82		0.52		0.93		0.8		0.62	
		Causes		Features		Treatment		Effect		Prevention	
Nationality		Good	Poor	Good	Poor	Good	Poor	Good	Poor	Good	Poor
Saudi	n	573	491	583	481	484	580	527	537	511	553
	%	53.9	46.1	54.8	45.2	45.5	54.5	49.5	50.5	48.0	52.0
Non-Saudi	n	57	78	69	66	43	92	50	85	55	80
	%	42.2	57.8	51.1	48.9	31.9	68.1	37.0	63.0	40.7	59.3
P-value		0.01*		0.42		0.0026**		0.0062**		0.11	
		Causes		Features		Treatment		Effect		Prevention	
City of residence		Good	Poor	Good	Poor	Good	Poor	Good	Poor	Good	Poor
Makkah	n	199	193	200	192	167	225	191	201	192	200
	%	50.8	49.2	51.0	49.0	42.6	57.4	48.7	51.3	49.0	51.0
Jeddah	n	152	134	168	118	114	172	124	162	126	160
	%	53.1	46.9	58.7	41.3	39.9	60.1	43.4	56.6	44.1	55.9
Taif	n	119	118	126	111	106	131	112	125	111	126
	%	50.2	49.8	53.2	46.8	44.7	55.3	47.3	52.7	46.8	53.2
Medinah	n	160	124	158	126	140	144	150	134	137	147
	%	56.3	43.7	55.6	44.4	49.3	50.7	52.8	47.2	48.2	51.8
P-value		0.44		0.23		0.13		0.16		0.62	
		Causes		Features		Treatment		Effect		Prevention	
Marital status		Good	Poor	Good	Poor	Good	Poor	Good	Poor	Good	Poor
Single	n	392	354	420	326	320	426	359	387	344	402
	%	52.5	47.5	56.3	43.7	42.9	57.1	48.1	51.9	46.1	53.9
Married	n	215	196	205	206	186	225	199	212	201	210
	%	52.3	47.7	49.9	50.1	45.3	54.7	48.4	51.6	48.9	51.1
Divorced	n	13	15	16	12	12	16	10	18	12	16
	%	46.4	53.6	57.1	42.9	42.9	57.1	35.7	64.3	42.9	57.1
Widowed	n	10	4	11	3	9	5	9	5	9	5
	%	71.4	28.6	78.6	21.4	64.3	35.7	64.3	35.7	64.3	35.7
P-value		0.49		0.049*		0.39		0.36		0.44	
		Causes		Features		Treatment		Effect		Prevention	
Education		Good	Poor	Good	Poor	Good	Poor	Good	Poor	Good	Poor
Primary	n	4	3	4	3	3	4	4	3	3	4
	%	57.1	42.9	57.1	42.9	42.9	57.1	57.1	42.9	42.9	57.1
Intermediate	n	15	14	18	11	14	15	12	17	15	14
	%	51.7	48.3	62.1	37.9	48.3	51.7	41.4	58.6	51.7	48.3
Secondary	n	167	141	167	141	138	170	149	159	148	160
	%	54.2	45.8	54.2	45.8	44.8	55.2	48.4	51.6	48.1	51.9
University	n	408	386	425	369	341	453	376	418	366	428
	%	51.4	48.6	53.5	46.5	42.9	57.1	47.4	52.6	46.1	53.9
Postgraduate	n	36	25	38	23	31	30	36	25	34	27
	%	59.0	41.0	62.3	37.7	50.8	49.2	59.0	41.0	55.7	44.3
P-value		0.76		0.65		0.77		0.43		0.64	
		Causes		Features		Treatment		Effect		Prevention	
Occupation		Good	Poor	Good	Poor	Good	Poor	Good	Poor	Good	Poor
Student	n	336	300	367	269	271	365	309	327	293	343
	%	52.8	47.2	57.7	42.3	42.6	57.4	48.6	51.4	46.1	53.9
Professional	n	160	142	155	147	143	159	143	159	146	156
	%	53.0	47.0	51.3	48.7	52.6	47.4	47.4	52.6	48.3	51.7
Self-employed	n	15	9	14	10	11	13	10	14	13	11
	%	62.5	37.5	58.3	41.7	45.8	54.2	41.7	58.3	54.2	45.8
Retired	n	41	41	44	38	39	43	42	40	41	41
	%	50.0	50.0	53.7	46.3	47.6	52.4	51.2	48.8	50.0	50.0
Housewife	n	43	41	36	48	35	49	39	45	45	39
	%	51.2	48.8	42.9	57.1	41.7	58.3	46.4	53.6	53.6	46.4
Unemployed	n	35	36	36	35	28	43	34	37	28	43
	%	49.3	50.7	50.7	49.3	39.4	60.6	47.9	52.1	39.4	60.6
P-value		0.9		0.12		0.68		0.97		0.51	
		Causes		Features		Treatment		Effect		Prevention	
Family monthly income		Good	Poor	Good	Poor	Good	Poor	Good	Poor	Good	Poor
0-5000 SAR	n	126	131	135	122	110	147	119	138	124	133
	%	49.0	51.0	52.5	47.5	42.8	57.2	46.3	53.7	48.2	51.8
5001-10000 SAR	n	105	118	105	118	92	131	99	124	100	123
	%	47.1	53.0	47.1	53.0	41.3	58.7	44.4	55.6	44.8	55.2
10001-15000 SAR	n	150	140	167	123	126	164	133	157	128	162
	%	51.7	48.3	57.6	42.4	43.4	56.6	45.9	54.1	44.1	55.9
15001-20000 SAR	n	123	95	118	100	95	123	107	111	104	114
	%	56.4	43.6	54.1	45.9	43.6	56.4	49.1	50.9	47.7	52.3
>20000 SAR	n	126	85	127	84	104	107	119	92	110	101
	%	59.7	40.3	60.2	39.8	49.3	50.7	56.4	43.6	52.1	47.9
P-value		0.046*		0.056		0.51		0.09		0.44	

As for nationality, Saudi participants are more aware of CTS than non-Saudis in all categories, ranging from 45.5% to 54.8% for Saudis and 31.9% to 51.1% for non-Saudis. The difference was statistically significant for causes, treatment, and effect. Furthermore, the level of awareness of different categories of CTS slightly varies across the four cities. Medinah had the highest overall percentage of good awareness (ranging from 48.2% to 56.3%), except for features and prevention, while Jeddah had better awareness of features, and Makkah had the highest levels of prevention awareness. Awareness levels ranged from 39.9% to 58.7% in Jeddah, 42.6% to 51.0% in Makkah, and 44.7% to 53.2% in Taif. However, this difference was not statistically significant between the four cities. In terms of awareness of CTS in the western region, there are slight differences between the cities involved. Medinah had the highest overall percentage of good awareness, ranging from 48.2% to 56.3%, except for features and prevention. Jeddah had better awareness of features, and Makkah had the highest levels of prevention awareness. The percentages of good awareness levels ranged from 39.9% to 58.7% in Jeddah, 42.6% to 51.0% in Makkah, and 44.7% to 53.2% in Taif. However, the differences were not statistically significant between the four cities.

As for education level, postgraduate students have the best awareness of CTS, with good awareness levels ranging from 50.8% to 62.3%. Participants at the university level also showed good awareness levels, ranging from 42.9% to 53.5%, while participants in secondary education had good levels ranging from 44.8% to 54.2%. Awareness levels for primary and intermediate education were not as high but still showed good levels, from 41.4% to 57.1%. However, the study did not find any statistically significant differences in awareness levels based on education level. Speaking of occupation, self-employed individuals had the highest awareness of the causes (62.5%), features (58.3%), and prevention (54.2%) of CTS. Meanwhile, professionals had the highest percentage of good awareness of the treatment of CTS (52.6%). Students also did quite well in terms of awareness levels among different categories of CTS. Unfortunately, the unemployed group had the lowest percentage of good awareness in most categories. However, there were no significant differences in the awareness levels between the occupation groups. Additionally, participants in the >20000 monthly income group showed the highest percentage of good awareness levels (59.7%), while the 5001-10000 group had the highest percentage of poor awareness (53.0%). Interestingly, there was a statistically significant association between family monthly income and awareness levels of the causes of CTS. As the family monthly income increases (>10001), there is a decrease in the percentage of participants with poor overall awareness and a decrease in the percentage of those with good overall awareness as we move toward the lowest income groups (<10001). However, no significant associations were found between income and CTS awareness.

Table 8 displays the distribution of chronic diseases and CTS. Out of the total participants, 136 (11.3%) had chronic diseases, while 1,063 (88.7%) did not. Among those who had chronic diseases, 27 (19.9%) had CTS, and 109 (80.1%) did not. Furthermore, 22.2% of people who had CTS also had chronic diseases, while 77.8% of people who had CTS did not have any chronic diseases. Although the p-value for the association is 0.07, it is not statistically significant, but there is some evidence of an association.

**Table 8:** Association between CTS and chronic diseases CTS: Carpal tunnel syndrome.

		Chronic diseases (n = 136)				Total (n = 1199)	P-values
		Yes		No			
CTS (n = 27)	Yes	6	22.2%	21	77.8%	27	0.07
	No	130	11.1%	1042	88.9%	1172	
Total		136		1063		1199	

## Discussion

The most prevalent peripheral nerve entrapment syndrome worldwide is CTS, which occurs due to the compression of the median nerve in the carpal tunnel at the wrist [13]. The purpose of this study is to evaluate the level of awareness and knowledge about CTS among the general population.

Our study findings indicate that the population surveyed has inadequate knowledge about CTS, particularly regarding its treatment, effects, and prevention methods. This supports the findings of the Al-Jouf region study, which reported a lack of awareness among most of the participants [11]. The lack of awareness surrounding CTS can be attributed to various factors. First, ensuring that accurate and comprehensive information about CTS is easily accessible to the public is essential. Healthcare providers and authorities can help by creating easily accessible educational resources, such as online materials and pamphlets. These resources should focus on spreading crucial information about CTS symptoms, available treatments, and prevention strategies. Empowering individuals with this knowledge will enable them to recognize early signs of CTS and seek appropriate medical attention promptly.

Additionally, cultural and societal beliefs may influence the level of awareness about CTS. In some communities, there might be a stigma associated with seeking medical help for musculoskeletal issues, causing individuals to overlook or neglect the symptoms of CTS. To address these cultural barriers, healthcare providers should collaborate with community leaders and organizations. By promoting open discussions about hand and wrist health, we can work toward overcoming these challenges and improving awareness about CTS.

Interestingly, our study identified a relatively better awareness of the causes and features of CTS (52.5% and 54.4%, respectively). This could be attributed to the visibility of some risk factors associated with CTS, such as repetitive hand motions in certain occupations. To capitalize on this relatively higher awareness, workplace interventions can be implemented in jobs involving repetitive hand movements, where ergonomic improvements and frequent breaks could help reduce the risk of CTS development.

However, our findings contrast with the study conducted in Al-Majmaah city, Saudi Arabia, which found that the level of awareness among the adult population was sufficient. The prevalence of CTS among our study participants (2%) may be attributed to the disparity in awareness levels between our study and Al-Majmaah's study, where the prevalence was much higher (14%) [7]. Notably, our study's CTS prevalence was slightly lower than that of a Swedish study, where it was approximately 4% [6].

In terms of the correlation between the level of CTS awareness and various factors, females showed marginally better awareness than males across all variables, although the difference was not statistically significant except for effect awareness. While further investigation is needed to fully understand the reasons behind this gender difference, it emphasizes the significance of involving both genders in focused awareness campaigns.

Younger age groups, Saudis, and postgraduate students tended to exhibit higher levels of CTS awareness. In the Al-Jouf region study, age was significantly associated with the level of awareness, with adults aged 18-30 having the best awareness in comparison to other age groups [11]. This may be related to greater exposure to health-related information through formal education and media consumption. To reach older age groups and individuals with lower education levels, healthcare providers could collaborate with elderly care centers, local community organizations, and schools to organize informative workshops and awareness sessions.

Self-employed individuals demonstrated greater awareness of the causes and features of CTS, which may be attributed to their increased exposure to activities involving repetitive hand motions, a known risk factor for CTS development. These findings have implications for healthcare providers designing public health campaigns and educational programs aimed at increasing CTS awareness and prevention. Specifically, targeting groups with lower awareness, such as males and older age groups, may help enhance overall awareness and decrease CTS incidence.

The most common chronic disease among participants in our study was DM, followed by hypothyroidism, RA, and amyloidosis. This is exactly similar to the Al-Majmaah study regarding the prevalence of chronic diseases. Our study suggests that there is some evidence that chronic illnesses and CTS are related (p = 0.07), while the Al-Majmaah study found a strong link between chronic illnesses and CTS, where approximately 30% of the participants who were affected by at least one of the chronic diseases had CTS at the same time [7]. Therefore, it is crucial to screen CTS patients for the presence of the previously mentioned chronic illnesses.

Limitations

Despite making an effort to get representative results, this study encountered several obstacles that led to some limitations. While the survey was performed online, physical forms would have provided more reliable results. Additionally, the majority of respondents were Saudi (88.7%), single (62.2%), with a university level of education (66.2%), and students (53%). Furthermore, only 2% of study participants had CTS, which may be a factor in the low awareness levels. These situations may have led to unintentional biases in the findings. However, more research is required in this field, utilizing various approaches, concentrating on various sociodemographic traits, and looking into other understudied areas of Saudi Arabia.

## Conclusions

The study demonstrates that people have inadequate awareness of CTS, particularly in regard to its effects, treatment, and prevention. However, they are more aware of the causes and features. The findings imply that healthcare providers should create focused public health campaigns and educational programs to raise awareness of CTS, especially among populations with low awareness (older age groups and males). The study also emphasizes a potential link between CTS and chronic illnesses, suggesting screening CTS patients for the existence of such conditions. These findings are helpful in improving patient outcomes and lowering the incidence of CTS.
